# 

**Published:** 1897-07

**Authors:** 


### NOTICES.

### The Southern Dental Association.

The Southern Dental Association will hold its next meeting, the 28th annual meeting of said Association, at Old Point Comfort, Va., August 3d to 6th, inclusive, headquarters at the Hygeia Hotel. A fine programme is being prepared and all members are urgently requested to be present. The next meeting will doubtless be one of the most important in the history of the

Association, and all members are expected to be on hand, as at this meeting the question of consolidation with the American will be settled. S. W. Foster, *Recording Secretary.*

### The National Association of Dental Examiners.

The fourteenth annual session of the National Association of Dental Examiners will be held in the parlors of the "Hotel Chamberlin," Old Point Comfort, Va., Friday morning, July 30th, 1897, ten o'clock, and continue in session (with the exception of Sunday, August 1st) until adjournment. Matters of the greatest importance to the Association will come before this meeting, and it is hoped every member of all the various State Boards will be present.

"Hotel Chamberlin" and "Hygeia Hotel" rates will be $2.50 per day, two in a room; $3.50 per day, one in a room.

Fare from all points on all the railroads will be one and a third; take your receipt from ticket agent when purchasing ticket. The Old Dominion Steamship Co., Pier 26, North River, New York, will sell excursion tickets, including meals and berth, for $11.20, sailing Thursday, July 29th, 3 p. m.

Charles A. Meeker, D. D. S., *Secretary*,
29 Fulton Street, Newark, N. J.

### American Dental Association.

The American Dental Association will hold its next meeting at Old Point Comfort, Va., Tuesday, August 3, 1897. This will, probably, be the most important meeting of the American Dental Association held in years, as it is expected that the entire question of reorganization will be presented for settlement. It is therefore earnestly desired that each organization in affiliation with the American will make a responsive effort to have a full delegation present at Old Point Comfort, and have this representative body instructed in regard to the position held by your society in relation to this question.

It is further suggested that each society should devote at least one evening to the discussion of the question: Whether a change in the relations of the two so-called national bodies, the American Dental Association and the Southern Dental Association, be desirable? In this way thought may be crystalized, and each delegate be prepared to meet the subject with the intelligence its importance demands.

Each State and local society which has adopted substantially the same code of ethics as that governing the conduct of members of the American Dental Association is entitled to one representative for every five members and fractional part thereof.

Blank certificates for delegates may be had on application to the corresponding secretary.

By order of the President, Dr. James Truman.

EMMA EAMES CHASE,
*Corresponding Secretary American Dental Association.*

### EDITORIAL.

### Twelfth International Medical Congress.

This body will be held in Moscow, Russia, August the 19th to 26th, 1897. In arranging the sections and the program for this meeting, the section on Dental Surgery appears not to have the position accorded to it in former Congresses. This may be owing to conditions and circumstances that cannot be appreciated or fully understood by those located so remotely from the place of the meeting. We are glad to see, however, that the section on Odontology has been assigned a place; it is the 9a section.

A program of subjects has been prepared and presented for the work of this section. The subjects are all interesting and doubtless will have careful and thorough consideration. The program is as follows:

1. What kind of general and special learning is desirable for the persons, who are to occupy themselves with Dentistry? The lecturer: Professor Dr. Julius Scheff, of Vienna.

2. The Hygiene of the cavity of the mouth and of the teeth.

3. General and local anæsthetics for tooth extraction. The lecturer: Dr. V. Richardson, of London.

4. Kataphoresis in Dentistry.

5. The essence and treatment of Pyorrhoea alveolaris. The lecturer: Prof. Dr. Jozsef Arkovy, of Budapest.

6. The treatment and filling of the pulpless teeth.

7. Crown and bridgework from the hygienic and technical point. The lecturer: M. Morgenstern, of Baden-Baden.

